# Vitamin D Decreases Serum VEGF Correlating with Clinical Improvement in Vitamin D-Deficient Women with PCOS: A Randomized Placebo-Controlled Trial

**DOI:** 10.3390/nu9040334

**Published:** 2017-03-28

**Authors:** Mohamad Irani, David B. Seifer, Richard V. Grazi, Sara Irani, Zev Rosenwaks, Reshef Tal

**Affiliations:** 1 The Ronald O. Perelman and Claudia Cohen Center for Reproductive Medicine, Weill Cornell Medicine, New York, NY 10021, USA; moi9010@med.cornell.edu; 2 Dartmouth-Hitchcock Medical Center, Geisel School of Medicine at Dartmouth, Lebanon, NH 03756, USA; dseifer@comcast.net; 3 Genesis Fertility and Reproductive Medicine, Maimonides Medical Center, Brooklyn, NY 11204, USA; rgrazi@genesisfertility.com; 4 Molecular and Cell Biology Program, School of Graduate Studies and Departments of Cell Biology and Pediatrics, State University of New York (SUNY) Downstate Medical Center, Brooklyn, NY 11203, USA; sarah.irani@hotmail.com; 5 Division of Reproductive Endocrinology and Infertility, Department of Obstetrics, Gynecology and Reproductive Sciences, Yale University School of Medicine, New Haven, CT 06520, USA; resheft@gmail.com

**Keywords:** vitamin D, vascular endothelial growth factor, VEGF, polycystic ovary syndrome, PCOS

## Abstract

Vascular endothelial growth factor (VEGF) has been suggested to play a role in the pathophysiology of polycystic ovary syndrome (PCOS) and may contribute to increased risk of ovarian hyperstimulation syndrome (OHSS) in affected individuals. Vitamin D (VitD) supplementation improves multiple clinical parameters in VitD-deficient women with PCOS and decreases VEGF levels in several other pathologic conditions. Unveiling the basic mechanisms underlying the beneficial effects of vitamin D on PCOS may enhance our understanding of the pathophysiology of this syndrome. It may also suggest a new treatment for PCOS that can improve it through the same mechanism as vitamin D and can be given regardless of vitamin D levels. Therefore, we aimed to explore the effect of VitD supplementation on serum VEGF levels and assess whether changes in VEGF correlate with an improvement in characteristic clinical abnormalities of PCOS. This is a randomized placebo-controlled trial conducted between October 2013 and March 2015. Sixty-eight VitD-deficient women with PCOS were recruited. Women received either 50,000 IU of oral VitD3 or placebo once weekly for 8 weeks. There was a significant decrease in serum VEGF levels (1106.4 ± 36.5 to 965.3 ± 42.7 pg·mL^–1^; *p* < 0.001) in the VitD group. Previously reported findings of this trial demonstrated a significant decrease in the intermenstrual intervals, Ferriman-Gallwey hirsutism score, and triglycerides following VitD supplementation. Interestingly, ∆VEGF was positively correlated with ∆triglycerides (*R*^2^ = 0.22; *p* = 0.02) following VitD supplementation. In conclusion, VitD replacement significantly decreases serum VEGF levels correlating with a decrease in triglycerides in women with PCOS. This is a novel molecular explanation for the beneficial effects of VitD treatment. It also suggests the need to investigate a potential role of VitD treatment in reducing the incidence or severity of OHSS in VitD-deficient women with PCOS.

## 1. Introduction

Polycystic ovary syndrome (PCOS) is a common endocrinopathy affecting 6%–10% of reproductive-aged women [1,2]. It is characterized by menstrual dysfunction, subfertility, polycystic ovaries and hyperandrogenism [3]. PCOS is also associated with an increased risk of depression, anxiety, endometrial carcinoma, cardiovascular disease, and multiple metabolic disorders such as insulin resistance, type II diabetes mellitus, dyslipidemia, high blood pressure and fatty liver [2,3].

The pathogenesis of PCOS is not well understood, but accumulating evidence suggests that vascular endothelial growth factor (VEGF) dysregulation may be play a role in its genesis [4]. VEGF is an approximately 46-kDA heparin-binding homodimeric protein that exists in six different isoforms: VEGF-A, VEGF-B, VEGF-C, VEGF-D, VEGF-E and placental growth factor [5,6,7]. It is a leading regulator of angiogenesis; indeed, its essential role has been demonstrated in physiological, developmental and pathological angiogenesis [8]. VEGF is a robust mitogen primarily for vascular endothelial cells [5]; it acts by binding to tyrosine kinase receptors, KDR (kinase domain region) and Flt-1 (fms-like-tyrosine kinase) receptors [8]. Ovaries of women with PCOS manifest upregulation of VEGF, associated with increased vascularity as measured by ultrasound Doppler blood flow and confirmed by histologic studies [9,10]. In addition, the hyperthecotic stroma of these ovaries overexpresses VEGF [11]. Furthermore, it has been shown that women with PCOS exhibit increased VEGF levels in serum and/or follicular fluid [12,13]. VEGF dysregulation in women with PCOS has also been correlated with an increased risk of ovarian hyperstimulation syndrome (OHSS) following follicular stimulation [14].

Vitamin D deficiency is more prevalent among women with PCOS when compared to controls [15]. The aforementioned deficiency has been associated with increased hirsutism score, insulin resistance and body mass index (BMI) in these women [16]. Additionally, vitamin D supplementation has been shown to improve blood pressure profiles and decrease insulin resistance, total testosterone and androstenedione levels in vitamin D-deficient women with PCOS [17,18]. However, the basic mechanisms underlying the beneficial effects of vitamin D in PCOS are still obscure. Resolving this mechanism may provide insight into the pathophysiology of this syndrome. It can also offer a new therapeutic option for PCOS women with normal vitamin D levels such as a medication that can improve PCOS through the same mechanism as vitamin D.

Interestingly, VEGF has been widely implicated in the pathogenesis of diabetes; for example, a negative correlation has been described between serum VEGF and vitamin D levels in diabetic patients [19,20]. In addition, vitamin D administration has been shown to decrease VEGF production by human lumbar annulus cells and various human cancer cells [21,22]. Thus, it could be speculated that vitamin D may ameliorate PCOS symptoms by regulating VEGF. Taken together, vitamin D supplementation in vitamin D-deficient women with PCOS could reduce serum VEGF levels, thus improving PCOS characteristic clinical manifestations. Of note, this is an extension of our previous work in the same patient cohort showing that transforming growth factor-β1 bioavailability decreases following vitamin D treatment [23].

## 2. Materials and Methods

### 2.1. Study Subjects

This study was a randomized, single-blind, placebo-controlled trial designed to assess the impact of vitamin D supplementation on serum VEGF levels and PCOS characteristic clinical manifestations in vitamin D-deficient women with PCOS [23]. All participants signed an informed consent form at the time of recruitment. The institutional review board (IRB) of Maimonides Medical Center approved the study. Ninety-three reproductive-aged women (18–38 years) diagnosed with PCOS according to the Rotterdam criteria presented to Maimonides Women’s Health center for well-woman visit between October 2013 and March 2015 were screened for vitamin D deficiency [24]. Women were considered vitamin D-deficient when their serum 25 hydroxy-vitamin D (25OH-D) levels were less than 20 ng·mL^–1^. We excluded women who were: (1) pregnant, postpartum or breastfeeding; (2) taking metformin, vitamin D supplements or any hormonal therapy.

### 2.2. Interventions and Blood Collection

A total of 68 women with PCOS were diagnosed with vitamin D deficiency. Participants were randomly allocated using a ratio of 2/1 (vitamin D/placebo) into the following two arms: (1) 50,000 IU of vitamin D3 once weekly for 8 weeks as per the Endocrine Society guidelines [25] and (2) placebo once weekly for 8 weeks. The placebo capsule looked similar to the vitamin D capsule but contained only lactose monohydrate powder (Gallipot Inc., Saint Paul, MN, USA). All participants were contacted once weekly and reminded to take their pill. Fasting blood samples were collected by venipuncture in both arms before starting and within two weeks of completing the treatment. Blood samples were allowed to clot for 30 min at room temperature before centrifugation at 1200 rpm for 10 min. Serum was stored in aliquots at −80 °C until assayed.

### 2.3. Assays of All Measured Hormones, 25OH-D and VEGF

Serum 25OH-D levels were measured before starting treatment and after study completion. Assays for 25OH-D were performed using the ADVIA Centaur vitamin D assay (Siemens Healthcare Diagnostics, Malvern, PA, USA). Testosterone, sex hormone-binding globulin (SHBG), thyroid-stimulating hormone (TSH), dehydroepiandrosterone sulfate (DHEAS), follicle-stimulating hormone (FSH), and luteinizing hormone (LH) were quantified using IMMULITE 2000 XPi immunoassay system (Siemens Healthcare Diagnostics). DXL 800 immunoassay analyzer was utilized to measure insulin and prolactin concentrations according to manufacturer’s protocols (Beckman Coulter Inc., Brea, CA, USA). The homeostatic model assessment (HOMA) was employed to calculate insulin resistance according to the following formula: (fasting insulin (µU·mL^–1^) × fasting glucose (mmol·L^–1^))/22.5 [26]. 17OH-progesterone level was measured by ELISA assay (Eagle BioSciences Inc., Nashua, NH, USA). VEGF concentration was quantified using Human VEGF Quantikine ELISA kit according to manufacturer’s protocols (R&D Systems, Minneapolis, MN, USA). The intra-assay and inter-assay coefficients of variation for all assays were less than 10%.

### 2.4. Clinical Parameters

The clinical parameters related to PCOS were assessed before and two months after the completion of treatment. These parameters included Ferriman-Gallwey score (FGS) (hirsutism score), acne status, blood pressure (BP), and intermenstrual intervals.

### 2.5. Statistical Analysis

Data were tested for normality. All values were expressed as mean ± standard error of the mean (SEM). A paired student’s *t*-test was used to compare the clinical parameters and serum levels evaluated before and after completing the treatment. Correlations between changes in serum VEGF and changes in disease clinical parameters were analyzed using Pearson’s test and linear regression. All data analyses were performed with STATA statistical software version 14 (StataCorp LP, College Station, TX, USA). *p* < 0.05 was considered to be statistically significant.

## 3. Results

### 3.1. Demographics and Changes in Serum 25OH-D Levels

As previously described, 53 participants completed the study: 35 in the vitamin D group and 18 in the placebo group [23]. BMI was comparable between the vitamin D and placebo groups (30 ± 1 and 28 ± 1.6 kg·m^–2^ respectively, *p* = 0.33). Women in both groups had comparable demographic characteristics such as age, skin color, ethnicity, smoking, daily milk consumption, and history of infertility (*p* ≥ 0.2). There was a significant increase in serum 25OH-D level reaching the normal range following vitamin D supplementation (16.3 ± 0.9 to 43.2 ± 2.4 ng·mL^–1^; *p* < 0.01) while it did not significantly change following placebo (17 ± 1.8 to 17.4 ± 1.9 ng·mL^–1^; *p* = 0.85) [23].

### 3.2. Changes in PCOS Clinical and Biochemical Parameters Following Vitamin D Supplementation

The previously reported findings of this trial showed a significant decrease in Ferriman-Gallwey hirsutism score (FGS) (9.8 ± 1.5 to 8.1 ± 1.5; *p* < 0.01), intermenstrual intervals (80 ± 9 to 60 ± 6 days; *p* = 0.04), and serum triglyceride levels (138 ± 22 to 117 ± 20 mg·dL^–1^; *p* = 0.03) after vitamin D supplementation [23]. However, there was no significant change in any of the other parameters measured (low density lipoprotein, high density lipoprotein, total cholesterol, DHEAS, free testosterone, FSH, LH, LH/FSH, fasting glucose, fasting insulin, HOMA-IR, systolic blood pressure, diastolic blood pressure, and mean arterial pressure) [23].

### 3.3. Changes in VEGF Following Vitamin D Supplementation

**Figure 1:**
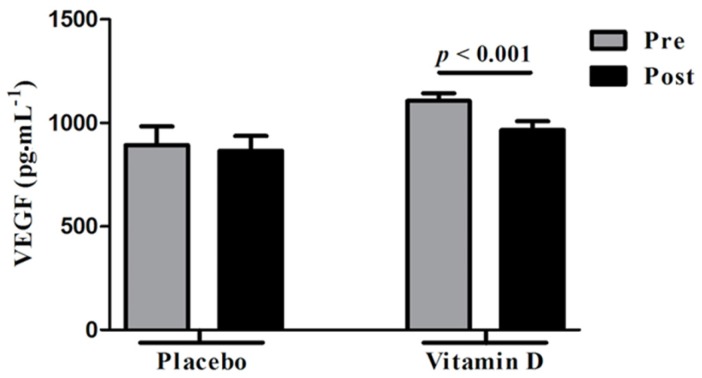
Changes in serum vascular endothelial growth factor (VEGF) levels following placebo and vitamin D3 supplementation. Vitamin D supplementation significantly decreased serum VEGF levels in vitamin D-deficient women with polycystic ovary syndrome (PCOS). There was no significant change in serum VEGF levels after placebo. Pre, before receiving vitamin D or placebo; Post, following supplementation with vitamin D or placebo.

There was a significant decrease in serum VEGF levels (1106.4 ± 36.5 to 965.3 ± 42.7 pg·mL^–1^; *p* < 0.001) in the vitamin D group, but not in the control group (893.1 ± 90.2 to 866 ± 70.8 pg·mL^–1^; *p* = 0.83) (Figure 1).

**Figure 2:**
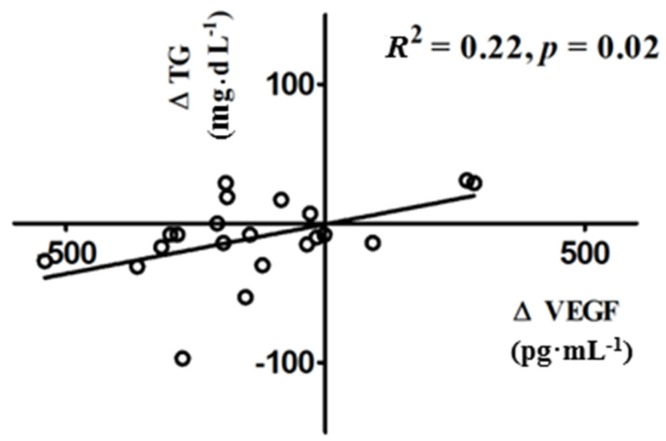
Correlation between ∆VEGF and ∆triglycerides following vitamin D3 supplementation. Following vitamin D replacement the decrease in VEGF positively correlated with the decrease in triglycerides in vitamin D-deficient women with PCOS.

Correlation and linear regression analyses were used to evaluate the relationship between the change in serum VEGF and changes in clinical and/or biochemical parameters in women with PCOS. The decrease in serum VEGF levels was positively correlated with the decrease in triglycerides (*R*^2^ = 0.22; *p* = 0.02) following vitamin D supplementation (Figure 2). The decrease in VEGF was not correlated with the decrease in FGS (*p* = 0.25), intermenstrual intervals (*p* = 0.7), or any other PCOS clinical or biochemical parameters.

## 4. Discussion

The current study examined the effect of vitamin D supplementation on serum VEGF levels and its correlation with the changes in PCOS clinical and biochemical manifestations in vitamin D-deficient women with PCOS. Our data show that vitamin D supplementation decreases serum VEGF levels and this change positively correlates with the decrease in triglycerides. 

Our findings, showing a significant decrease in VEGF following vitamin D treatment, are consistent with multiple previous studies [19,21,22,27,28]. In fact, Shao et al. have recently shown that serum 25OH-D3 negatively correlates with VEGF in diabetic patients; such correlation suggests that the protective effects of vitamin D in terms of decreasing proteinuria and delaying the progression of diabetic kidney disease may be mediated through its suppression of abnormal angiogenesis, inflammation, and vascular endothelial dysfunction [19]. Moreover, Ren et al. have confirmed that 1,25(OH)_2_-D3 downregulates the expression of VEGF in the retinal tissues of diabetic rats, thereby protecting them against diabetic retinopathy [28]. Similarly, Yildirim et al. have demonstrated that 1,25(OH)_2_-D3 regresses endometriotic implants in rat models by impeding the expression of VEGF in these implants, thus inhibiting inflammation and neovascularization [27]. Likewise, Ben-Shoshan et al. have shown that 1,25(OH)_2_-D3 inhibits VEGF expression in various human cells (breast, colon, and prostate) under normoxic and hypoxic conditions [21]. Gruber et al. have also shown that 1,25(OH)_2_-D3 decreases the production of VEGF in human lumbar annulus cells [22]. 

There is compelling evidence suggesting an important role of VEGF in the pathophysiology of PCOS [4,11,12,13,14,29]. VEGF, the prototypical member of the angiogenic factors, may be implicated in the increased ovarian mass supported by excessive neovascularization in stroma and theca of PCOS ovaries. Serum levels of VEGF have been reported to be elevated in women with PCOS compared with normal women [12,29]. VEGF levels are also elevated while its circulating receptor Flt-1 levels are decreased in the follicular fluid of women with PCOS undergoing controlled ovarian hyperstimulation compared with controls, which may explain their increased risk of ovarian hyperstimulation syndrome [13,14,30,31]. Furthermore, PCOS ovaries overexpress VEGF mRNA particularly in hyperthecotic stroma cells and some follicular granulosa cells [11]. In addition, endocrine gland-VEGF, which is an endothelial cell mitogen with selectivity for endothelium of steroidogenic glands, has been shown to be overexpressed in the theca interna and stroma of PCOS ovaries [32]. Our data showing that the decrease in VEGF after vitamin D treatment is correlated with a decrease in triglycerides are in line with the literature supporting the role of VEGF in the pathogenesis of PCOS. 

Vitamin D treatment has been shown to improve the characteristic clinical manifestations of PCOS in vitamin D-deficient women [17,18,23,33]. Selimoglu et al. have shown that the administration of a single dose of 300,000 IU of vitamin D3 was associated with a significant decrease in insulin resistance [18]. Pal et al. have also shown that the daily administration of 8533 IU of vitamin D and 530 mg of elemental calcium for 3 months was associated with a significant reduction in blood pressure parameters and total testosterone levels [17]. Furthermore, vitamin D3 supplementation with 50,000 IU once weekly for 8 weeks improved hirsutism, acne, and decreased triglycerides and intermenstrual intervals in women with PCOS [23]. The fact that vitamin D also decreases VEGF, correlating with an improvement in triglycerides levels supports the theory that the beneficial effects of vitamin D may be partly exerted through decreasing VEGF and subsequently inhibiting abnormal ovarian angiogenesis. Additionally, laparoscopic ovarian drilling in women with PCOS has been suggested to exert its effects via decrease in VEGF and associated abnormal ovarian vasculature [34]. However, in order to gain further insight into the mechanism underlying the beneficial effects of vitamin D in PCOS, in-depth molecular studies on vitamin D’s effects on human ovarian cells in culture as well as PCOS animal models are warranted.

The main limitation of this trial was its failure to adjust for the potential impact of seasonal variation on vitamin D levels. The seasonal changes and the skin’s exposure to sun can influence the skin’s production of vitamin D3 [25]. 

In conclusion, we have demonstrated that vitamin D supplementation in vitamin D-deficient women with PCOS significantly decreases serum VEGF levels correlating with a significant decrease in serum triglycerides. These data suggest a possible molecular mechanism by which vitamin D mitigates PCOS symptoms. Our findings support the role of VEGF in the pathophysiology of PCOS. It also underscores the need to investigate a potential role of vitamin D treatment in the incidence or severity of ovarian hyperstimulation syndrome in women with PCOS undergoing follicular stimulation.
